# Neuroimaging markers of global cognition in early Alzheimer's disease: A magnetic resonance imaging–electroencephalography study

**DOI:** 10.1002/brb3.1197

**Published:** 2018-12-27

**Authors:** Markus Waser, Thomas Benke, Peter Dal‐Bianco, Heinrich Garn, Jochen A. Mosbacher, Gerhard Ransmayr, Reinhold Schmidt, Stephan Seiler, Helge B. D. Sorensen, Poul J. Jennum

**Affiliations:** ^1^ Biomedical Engineering Department of Electrical Engineering Technical University of Denmark Lyngby Denmark; ^2^ Danish Center for Sleep Medicine Department of Clinical Neurophysiology Rigshospitalet Glostrup Glostrup Denmark; ^3^ AIT Austrian Institute of Technology GmbH Center for Digital Safety & Security Sensing and Vision Solutions Vienna Austria; ^4^ Department of Neurology Medical University of Innsbruck Innsbruck Austria; ^5^ Department of Neurology Medical University of Vienna Vienna Austria; ^6^ Department of Neurology Medical University of Graz Graz Austria; ^7^ Clinic for Neurology II Kepler University Hospital Med Campus III Linz Austria

**Keywords:** Alzheimer disease, cognition, electroencephalography, magnetic resonance imaging

## Abstract

**Introduction:**

Magnetic resonance imaging (MRI) and electroencephalography (EEG) are a promising means to an objectified assessment of cognitive impairment in Alzheimer's disease (AD). Individually, however, these modalities tend to lack precision in both AD diagnosis and AD staging. A joint MRI–EEG approach that combines structural with functional information has the potential to overcome these limitations.

**Materials and Methods:**

This cross‐sectional study systematically investigated the link between MRI and EEG markers and the global cognitive status in early AD. We hypothesized that the joint modalities would identify cognitive deficits with higher accuracy than the individual modalities. In a cohort of 111 AD patients, we combined MRI measures of cortical thickness and regional brain volume with EEG measures of rhythmic activity, information processing and functional coupling in a generalized multiple regression model. Machine learning classification was used to evaluate the markers’ utility in accurately separating the subjects according to their cognitive score.

**Results:**

We found that joint measures of temporal volume, cortical thickness, and EEG slowing were well associated with the cognitive status and explained 38.2% of ifs variation. The inclusion of the covariates age, sex, and education considerably improved the model. The joint markers separated the subjects with an accuracy of 84.7%, which was considerably higher than by using individual modalities.

**Conclusions:**

These results suggest that including joint MRI–EEG markers may be beneficial in the diagnostic workup, thus allowing for adequate treatment. Further studies in larger populations, with a longitudinal design and validated against functional‐metabolic imaging are warranted to confirm the results.

## 1 INTRODUCTION

Alzheimer's disease (AD) is a fatal disorder that is associated with the accumulation of β‐amyloid plaques and neurofibrillary tau tangles causing progressive neurodegeneration in certain cortical and subcortical regions (Hyman et al., 2012). AD invariably affects episodic memory and other complex cognitive processes, but the perceived onset and early course of the symptoms are highly subjective and depend on the individual cognitive reserve (Stern, 2012). An accurate clinical assessment of the cognitive deficits is crucial for disease staging and thus for optimal pharmacological treatment and therapy planning. Typically, cognitive impairment is assessed in a doctor–patient/–caregiver interview and neuropsychological screening tests such as the mini‐mental state examination (MMSE; Folstein, Folstein, & McHugh, 1975). These tests, however, are susceptible to daily variations, and their outcome is affected by sociodemographic factors and the individual cognitive reserve (Crum, Anthony, Bassett, & Folstein, 1993). Consequently, there is a need for accurate alternatives to measure the progression of cognitive impairment in AD.

Magnetic resonance imaging (MRI) and electroencephalography (EEG) are potential surrogate in vivo measures of AD progression that are noninvasive, inexpensive, and widely available. MRI measures of regional brain volumes and cortical thickness are promising markers of both AD neuropathology and cognitive decline (Babiloni et al., 2015; Dubois et al., 2014; Scheltens, Fox, Barkhof, & De Carli, 2002). Especially, the limbic system in the medial temporal lobe has been identified to be vulnerable to AD: Typically, the entorhinal cortex is among the regions affected in the earliest disease stages (Killiany et al., 2002). The hippocampal volume appears to be a good marker of both disease onset and its progression (Bateman et al., 2012; Den Heijer et al., 2010; Dickerson et al., 2001; Jack et al., 2010; Lo et al., 2011). Other affected brain regions include amygdala, posterior association cortex, and the cholinergic basal forebrain (Bottino et al., 2002; Braak & Braak, 1991; Dubois et al., 2014). The cortical atrophic topography is often in line with the clinical phenotype; subjects with verbal memory impairment, for instance, frequently exhibit early atrophy in the left temporal lobe (Johnson, Fox, Sperling, & Klunk, 2012).

Electroencephalography, on the other hand, is a promising tool to assess the AD‐related functional disintegration of large scale brain networks such as the default mode network during resting state (Dillen et al., 2017; Horn, Ostwald, Reisert, & Blankenburg, 2014). Visual resting state EEG analyses in AD patients have revealed a slow dominant posterior rhythm and an increase in widespread delta and theta activity combined with a reduction in alpha and beta (Berger, 1937; Brenner, Reynolds, & Ulrich, 1988; Gordon & Sim, 1967; Letemendia & Pampiglione, 1958; Liddell, 1958; Rae‐Grant et al., 1987; Soininen, Partanen, Helkala, & Riekkinen, 1982; Weiner & Schuster, 1956). These abnormal EEG patterns have shown correlations with the cognitive status as well (Brenner et al., 1988; Gordon & Sim, 1967; Johannsen, Jakobsen, Bruhn, & Gjedde, 1999; Kaszniak, Garron, Fox, Bergen, & Huckman, 1979; Liddell, 1958; Merskey et al., 1980; Mundy‐Castle, Hurst, Beerstecher, & Prinsloo, 1954; Obrist, Busse, Eisdorfer, & Kleemeier, 1962; Rae‐Grant et al., 1987; Roberts, McGeorge, & Caird, 1978; Weiner & Schuster, 1956). Computerized resting state EEG studies have confirmed these early studies; they used the spectral power in predefined frequency bands to quantify EEG rhythmicity, synchrony‐measures such as coherence to quantify EEG connectivity, and measures from information theory to quantify EEG complexity (see Jeong, 2004 and Dauwels, Vialatte, & Cichocki, 2010 for extensive reviews). Besides resting state analyses, growing evidence suggests that the EEG recorded during memory encoding tasks carries essential information about other AD‐affected large‐scale brain networks (Garn et al., 2014, 2015; Hidasi, Czigler, Salacz, Csibri, & Molnár, 2007; Hogan, Swanwick, Kaiser, Rowan, & Lawlor, 2003; Jiang, 2005; Jiang & Zheng, 2006; Klimesch, Sauseng, & Hanslmayr, 2007; Pijnenburg et al., 2004; Stam, 2000; Stam, van Cappellen van Walsum, & Micheloyannis, 2002; Van der Hiele et al., 2007; Waser et al., 2016). With this in mind, EEG measures such as upper alpha desynchronization and theta synchronization during memory encoding might be the potential markers of impaired memory performance (Klimesch, 1999).

Despite their evident potential as noninvasive assistive tools in AD diagnose, MRI is routinely used solely for the purpose of excluding other possible causes such as vascular lesions, strategic lunar infarcts, or cerebral haemorrhages (Dubois et al., 2014; Schmidt et al., 2010), and EEG is not included in the standard diagnostic workup at all (American Psychiatric Association, 2013; Dubois et al., 2007, 2010, 2014; Hyman et al., 2012; McKhann et al., 1984, 2011; Schmidt et al., 2010). A reason is that, individually, these modalities tend to lack precision in both AD diagnosis and staging. However, combining the structural and functional information from MRI and EEG in a multimodal approach has the potential to overcome the limitations of the individual modalities. This cross‐sectional study systematically investigates the usefulness of different MRI and EEG measures as symptom‐independent markers of the cognitive deficits in a cohort of early AD patients. We hypothesize that (a) these markers are significantly related to global cognition as measured by MMSE in early AD and that (b) joint MRI–EEG markers allow for an accurate identification of significant cognitive deficits.

## 2 MATERIALS AND METHODS

### 2.1 Ethics statement

This research was approved by the ethics committees of the Medical Universities Graz, Innsbruck, Vienna and the Ethics Committee of the State of Upper Austria.

### 2.2 Study cohort

The study cohort consisted of 111 subjects diagnosed with probable (*N* = 77) or possible (*N* = 34) AD according to NINCDS‐ADRDA^1^ criteria (McKhann et al., 1984) who participated in the prospective dementia (PRODEM) study of the Austrian Alzheimer Society (Seiler et al., 2012). Enrollment criteria included the availability of a caregiver, written informed consent of participant and caregiver, no need for 24‐hr care, and the absence of other physical or neurological causes of dementia‐like symptoms. All patients underwent a routine laboratory assessment, measurement of serum vitamin B12 and folic acid levels, as well as serologic (HIV, Lues) and thyroid testing. AD‐inconsistent patterns of cerebral atrophy and lesion were excluded in T1‐weighted MRI (3D MPRAGE) sequences. The dementia severity was assessed by clinical dementia rating (CDR) on a scale of 0 (no dementia) to 3 (severe dementia) (Hughes, Berg, Danziger, Coben, & Martin, 1982; Morris, 1993). Our cohort consisted of subjects with CDR scores of 0.5 (very mild dementia) or 1 (mild dementia). The global cognition was assessed by MMSE scores on a scale of 0–30 where scores below 24 indicate significant cognitive deficits (Folstein et al., 1975; Mungas, 1991). The participants’ age, sex, and completed years of education were included as potential confounders (Crum et al., 1993; Kittner et al., 1986; O'Connor, Pollitt, Treasure, Brook, & Reiss, 1989).

### 2.3 MRI assessment

The brain structure was assessed by T1‐weighted whole‐brain MRI scanning (3D MPRAGE sequence) on one of the three different systems (Avanto 1.5 T, Symphony Tim 1.5 T and Trio Tim 3.0 T, all were manufactured by Siemens Healthcare, Erlangen, Germany). The selected sequence parameters were echo time 3.0 ms (1.9–4.2 ms), repetition time 2,020 ms (1,410–2,300 ms), inversion time 1,017 ms (800–1,100 ms), flip angle 9°, 10° and 15°, resolution 0.9 mm (0.83–1 mm) × 0.9 mm (0.83–1 mm) × 1 mm (1–1.2 mm), and bandwidth 130 Hz.

MRI scans were processed with the FreeSurfer neuroimaging software (Version 5.3, Massachusetts General Hospital, Boston MA, USA) that included automated methods for volume segmentation and measurement of cortical thickness of various brain regions. Briefly, the processing flow consisted of skull stripping followed by segmentation of gray/white matter and mapping of different brain structures in Talairach space (Desikan et al., 2006; Fischl et al., 2002). A detailed technical description can be found, for example, in Fischl et al., 2002 and Reuter, Schmansky, Rosas, & Fischl, 2012. In previous studies, these morphometric FreeSurfer procedures were validated against manual mapping (Fischl et al., 2002; Kuperberg et al., 2003; Salat et al., 2004) and proved to be reliable across scanner types and field strengths (Han et al., 2006; Reuter et al., 2012).

The regional volume and mean cortical thickness of the frontal, parietal, left and right temporal, and occipital lobe of the cerebral cortex – the major control of higher cognitive function – were employed as potential markers of cognitive impairment. The left and right temporal lobes were hereby assessed separately since the left temporal lobe has been described to be more affected in early AD stages (Johnson et al., 2012). In addition, we included the volume of entorhinal cortex, hippocampus, and amygdala due to their vulnerability to atrophy in early AD (Juottonen, Laakso, Partanen, & Soininen, 1999; Poulin, Dautoff, Morris, Barrett, & Dickerson, 2011). All MRI measures were normalized by the individual total intracranial volume to account for anatomical differences between the subjects (Fischl, 2012).

### 2.4 EEG assessment

The brain function was assessed by EEG recordings collected from 19 gold cup electrodes (Fp1, Fp2, F7, F3, Fz, F4, F8, T7, C3, Cz, C4, T8, P7, P3, Pz, P4, P8, O1, and O2; ground between Fz and Cz; connected mastoids as reference) placed according to the international 10–20 system (Jasper, 1958). Vertical and horizontal EOG and wrist‐ECG were recorded in parallel. All clinics used identical EEG systems (AlphaEEG amplifier by Alpha Trace Medical Systems, Vienna, Austria with NeuroSpeed software, bandpass 0.3–70 Hz (3 dB), notch 50 Hz, sampling rate 256 Hz, and resolution 16 bits). Impedances were kept below 10 kOhm.

The recordings were conducted in quiet and separated rooms with soft light in accordance with a predefined paradigm. Participants sat upright in comfortable chairs with neck support and a monitor positioned in front of them. They were asked to reduce movements to a minimum. A recording session included a 30‐s resting phase with closed eyes (REC) and a 30‐s memory encoding test with open eyes (ENC) where on‐screen face–name combinations had to be memorized. This test was designed to capture AD‐specific visual‐verbal memory deficits. The details of the paradigm were described elsewhere (Garn et al., 2015).

We used Matlab software (Version R2016b, MathWorks, Natick MA, USA) to remove non‐neuronal artifacts from the EEG. More specifically, each recording was downsampled to 128 Hz to reduce computational cost and bandpass filtered in the range of 1–30 Hz (type 1 FIR filter, 60 dB). Eye artifacts were removed by constrained independent component analysis using the EOG (Lu & Rajapakse, 2006). Cardiac artifacts were corrected by a modified Pan‐Tompkins algorithm using the ECG (Waser & Garn, 2013). The resulting EEG samples were divided in 2‐s epochs with 1‐s overlap (Blanco, Garcia, Quiroga, Romanelli, & Rosso, 1995). Epochs with residual artifacts were identified by a thresholding algorithm (Waser et al., 2017), visually validated by an EEG expert and omitted from further analyses.

**Figure 1:**
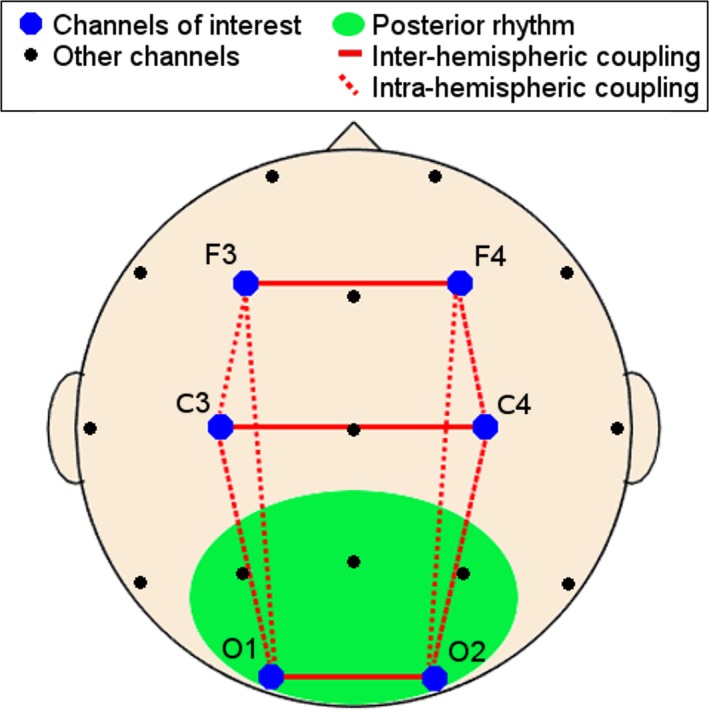
Electrode placement on the scalp as seen from above: The dominant posterior rhythm was measured in P3, Pz, P4, O1, and O2 (green area), whereas the remaining features were calculated in F3, F4, C3, C4, O1, and O2 (blue dots). Interhemispheric couplings are indicated by solid red lines and intrahemispheric coupling by dotted red lines

The EEG measures listed in Table 1 were computed epoch‐ and channel‐wise. Global markers were derived separately for REC and ENC by taking the average over all channels and epochs (cf. Table 1). A technical marker description can be found in Supporting information Material section A. In brief, the dominant posterior EEG rhythm was measured by the individual alpha frequency (IAF) defined as the mean frequency‐position of the spectral center of gravity between 8 and 13 Hz. Using the IAF as anchor frequency, the spectral power was computed in individualized frequency bands delta from IAF‐7 to IAF‐5 Hz, theta from IAF‐5 to IAF‐2 Hz, alpha1 from IAF‐2 to IAF Hz, alpha2 from IAF to IAF + 2 Hz, beta1 from IAF + 2 to IAF + 8 Hz and beta2 from IAF + 8 to IAF + 16 Hz. The auto‐mutual information (aMI) quantified the similarity of an EEG signal at different points in time and was designed to capture EEG complexity and information processing mechanisms (Jeong, Gore, & Peterson, 2001; Shannon & Weaver, 1949). Inter and intrahemispheric EEG connectivity was measured by coherence in a linear and by mutual information (cMI) in a nonlinear way. Figure 1 illustrates the topographic logic of the marker assessment.

**Table 1:** Summary of potential electroencephalography (EEG) markers

EEG markers	Channels	Assessment phase
Individual alpha frequency	P3, Pz, P4, O1, O2	Rest, eyes closed
Spectral delta‐power	F3, F4, C3, C4, O1, O2	Visual‐verbal encoding
Spectral theta power	F3, F4, C3, C4, O1, O2	Visual‐verbal encoding
Spectral alpha1 power	F3, F4, C3, C4, O1, O2	Visual‐verbal encoding
Spectral alpha2 power	F3, F4, C3, C4, O1, O2	Visual‐verbal encoding
Spectral beta1 power	F3, F4, C3, C4, O1, O2	Visual‐verbal encoding
Spectral beta2 power	F3, F4, C3, C4, O1, O2	Visual‐verbal encoding
Auto‐mutual information	F3, F4, C3, C4, O1, O2	Visual‐verbal encoding
Interhemispheric coherence	F3‐F4, C3‐C4, O1‐O2	Visual‐verbal encoding
Intrahemispheric coherence	F3‐C3, F3‐O1, C3‐O1, F4‐C4, F4‐O2, C4‐O2	Visual‐verbal encoding
Interhemispheric mutual info	F3‐F4, C3‐C4, O1‐O2	Visual‐verbal encoding
Intrahemispheric mutual info	F3‐C3, F3‐O1, C3‐O1, F4‐C4, F4‐O2, C4‐O2	Visual‐verbal encoding

### 2.5 Statistical analyses

Statistical analyses were performed with the R statistics software (Version 3.4.1, R Foundation for Statistical Computing, Vienna, Austria). We inspected the MMSE scores, covariates (age, sex, and years of education), as well as MRI and EEG markers in histograms, scatter‐ and boxplots. MMSE scores were log‐transformed to make them conform to normality. All data were rescaled to *z*‐scores with mean 0 and standard deviation 1. First, we tested the correlation of MMSE with each individual marker using *F*‐tests (α = 0.05). Second, a generalized multiple regression model was used to relate a combination of markers (regressors) to the cognitive scores (outcome) while accounting for age, sex, and education (covariates). More specifically, we used all‐subset selection to identify the marker subset that described the most MMSE variance in terms of *R* ^2^ value. To avoid overfitting, models with a large number of regressors were hereby penalized using the corrected Akaike information criterion (Akaike, 1973; Sugiura, 1978). The model assumptions were visually verified in diagnostic residual plots. The regression fit was tested by an overall *F*‐test and two‐tailed *t* tests of individual terms (α = 0.05). The central statistical concepts are described in detail in Supporting information Material Section B.

### 2.6 Diagnostic utility assessment

The same marker subset was then used to distinguish subjects with MMSE ≥ 24 from those with MMSE < 24 by a machine learning classification approach (see Supporting information Material Section B.3). We used a support vector machine (SVM) with radial basis function kernel to separate the two groups (Cortes & Vapnik, 1995). Parameters were hereby tuned using a grid search over defined parameter ranges. Classification was performed with leave‐one‐out cross‐validation and evaluated by its sensitivity (true positive rate), specificity (true negative rate), and accuracy. Group differences regarding age, sex, and years of education were tested by *χ*²‐ and Kruskal–Wallis tests.

## 3 RESULTS

### 3.1 Sample characteristics

Table 2 summarizes the demographic and clinical sample characteristics. The subjects’ age was negatively correlated with the cognitive scores (*r* = −0.22, *F*_(1,109)_ = 5.29, *p* = 0.023). There was, however, no significant age difference between the subjects with MMSE ≥ 24 and those with scores below 24. Neither the subjects’ sex nor their completed years of education were significantly related to the cognitive status. The rather weak MMSE–CDR relation ($\chi_{\left(1\right)}^{2}$ = 4.19, *p* = 0.041) in the earliest AD stages is in line with previous findings (Perneczky et al., 2006).

**Table 2:** Empirical and statistical sample description

	Total	Correlation with MMSE		MMSE ≥ 24	MMSE < 24	Difference
		Pearson *r*	*p* value			*p* value
Subject count	111			63	48	
Age (years)	74.6 ± 8.1	−0.22	0.023*	74.8 ± 7.2	74.3 ± 9.3	0.744
Sex (female)	61	−0.18	0.065	32	29	0.414
Education (years)	10.9 ± 2.9	0.16	0.091	11.2 ± 3.2	10.6 ± 2.4	0.253
MMSE	23.4 ± 3.1	‐	‐	25.5 ± 1.4	20.5 ± 2.3	‐
CDR (0.5 | 1)	73 | 38	−0.18	0.055	47 | 16	26 | 22	0.041*

*Note.* The *p* values in bold font with an asterisk (*) indicate statistical significance at alpha level 0.05.

### 3.2 Neuroimaging markers across the spectrum of cognitive impairment

#### 3.2.1 Individual markers

Table 3 lists the MRI and EEG markers and their respective (non‐normalized) mean values ± standard deviation. In relating individual markers with MMSE scores, we found significant results for the volumes of the parietal and the left temporal lobe, the spectral power in theta, alpha1, beta1, and beta2, as well as aMI. More specifically, reduced lobar volume, increased portions of spectral power in a low frequency range, and increased aMI were all associated with lower MMSE scores.

**Table 3:** Original magnetic resonance imaging (MRI) and electroencephalography (EEG) marker values (mean ± standard deviation) and linear regression analysis: The slope β refers to the linear regression coefficient of the normalized markers as regressors and log‐normalized MMSE scores as outcome, while correcting for age, sex, and completed years of education as covariates

Potential markers	Values	Regression analysis	
	Mean ± *SD*	Slope β	*p* value
MRI markers			
Cortical volumes [cm³]			
Frontal lobe	122.91 ± 17.41	0.053	0.579
Parietal lobe	77.31 ± 11.61	0.194	0.037*
Temporal lobe left	38.45 ± 6.22	0.188	0.045*
Temporal lobe right	38.48 ± 6.13	0.109	0.244
Occipital lobe	35.97 ± 5.48	0.126	0.176
Cortical thickness [mm]			
Frontal lobe	2.12 ± 0.22	0.137	0.144
Parietal lobe	1.78 ± 0.19	−0.009	0.921
Temporal lobe left	2.27 ± 0.30	−0.001	0.988
Temporal lobe right	2.34 ± 0.33	−0.034	0.721
Occipital lobe	1.60 ± 0.12	0.112	0.234
Limbic volumes [cm³]			
Entorhinal cortex	2.82 ± 0.67	0.080	0.393
Hippocampus	6.28 ± 1.19	−0.055	0.564
Amygdala	2.27 ± 0.55	0.058	0.543
EEG markers			
Posterior dominant rhythm in rest			
Individual alpha frequency	9.71 ± 0.44	0.093	0.328
Rhythmic activity			
Spectral delta power	0.11 ± 0.04	−0.176	0.057
Spectral theta power	0.15 ± 0.06	−0.380	0.001*
Spectral alpha1 power	0.09 ± 0.04	−0.220	0.018*
Spectral alpha2 power	0.07 ± 0.02	0.087	0.359
Spectral beta1 power	0.15 ± 0.04	0.284	0.002*
Spectral beta2 power	0.17 ± 0.07	0.225	0.014*
Information processing			
Auto‐mutual information	0.31 ± 0.01	−0.213	0.021*
Functional coupling			
Interhemispheric coherence	0.57 ± 0.09	0.177	0.058
Intrahemispheric coherence	0.41 ± 0.07	0.114	0.223
Interhemispheric mutual information	0.19 ± 0.01	−0.020	0.834
Intrahemispheric mutual information	0.17 ± 0.01	−0.037	0.691

Note

The *p* values in bold font with an asterisk (*) indicate statistical significance at alpha level 0.05.

*SD*: standard deviation.

**Figure 2:**
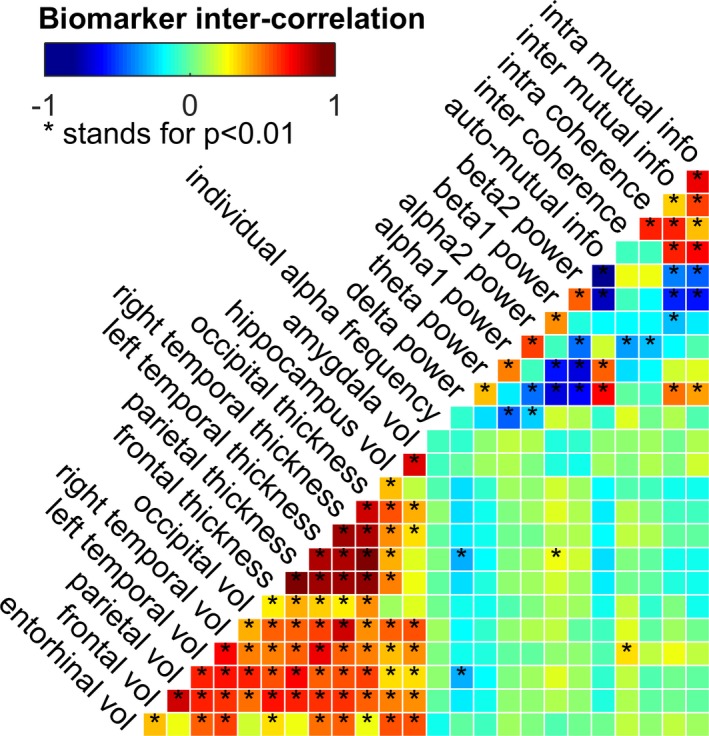
Analysis of marker intercorrelation: The Pearson's correlation is shown color‐coded and a (*) indicates a significant intercorrelation as tested by two‐tailed Student's *t* test (α = 0.01)

Figure 2 shows the between‐marker correlation (Pearson's *r*) color‐coded from blue (−1) to red (1) and tagged with a (*) in case of a high statistical significance (*p* < 0.01). We observed a widespread positive correlation between the MRI markers that were most pronounced in the cortical thickness markers. The relative spectral power in the delta and theta frequency bands was negatively correlated with the power in the higher beta1 and beta2 bands. The aMI showed high correlations with beta1 and beta2 power, indicating that information processing mechanisms were mainly reflected by a large portion of high‐frequency EEG oscillations. As for MRI–EEG correlations, reduced volume and cortical thickness of the parietal lobe were significantly associated with high delta‐power, the parietal volume was positively correlated with beta1 power as well as the left temporal volume with interhemispheric coherence.

#### 3.2.2 Marker combinations

**Figure 3:**
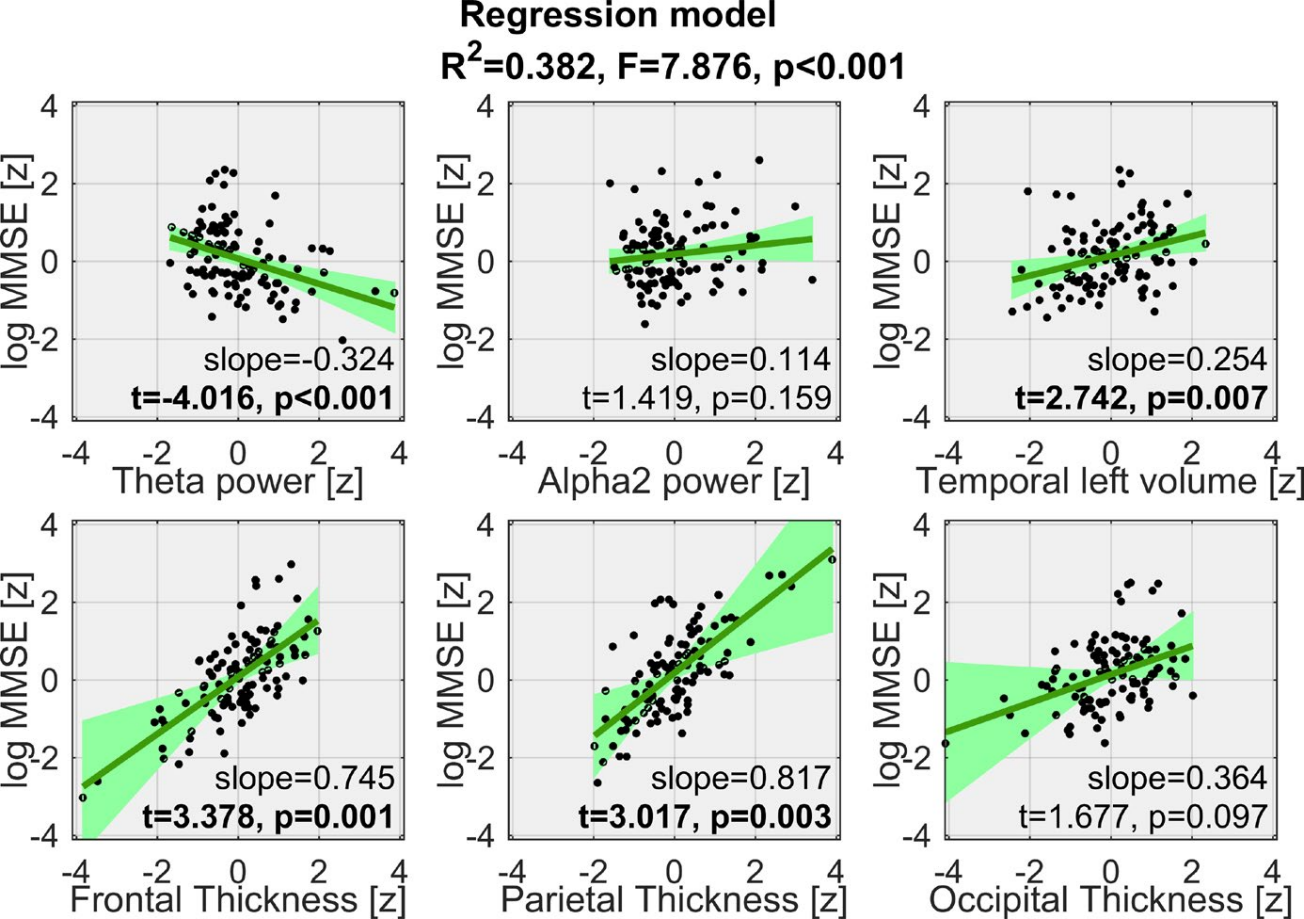
Visualization of the regression model: Each window shows the scatterplot of a standardized marker versus standardized log‐transformed MMSE scores (corrected for the remaining markers) where a black dot represents a subject, the green line represents the partial regression and the light green area its 95% confidence band. The combined markers explain 38.2% of MMSE variation

The following marker subset was selected to be included in the regression model: the left temporal volume, the cortical thickness of frontal, parietal and occipital lobe, and the spectral power in theta and alpha2. The regression was significant (*p* < 0.001) and the combined regressors explained 38.2% of the variation in MMSE scores. Figure 3 shows the relation of each regressor to the MMSE given that the remaining regressors were included in the same model. Among the individual effects, theta power (*p* < 0.001), left temporal volume (*p* = 0.007), frontal thickness (*p* = 0.001), and parietal thickness (*p* = 0.003) were significant with the latter two having the steepest slopes.

### 3.3 Diagnostic utility

**Figure 4:**
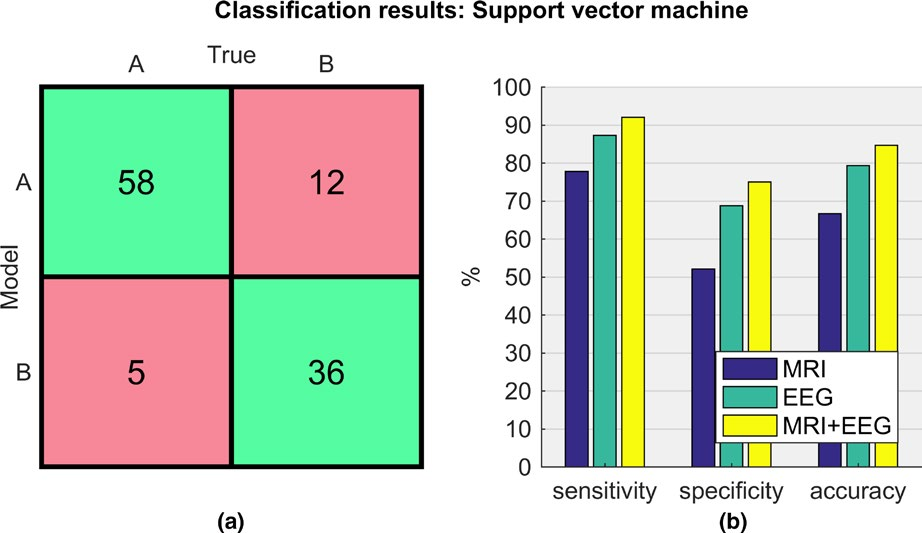
Classification results using a nonlinear approach: (a) Confusion matrix of the combined MRI–EEG classification containing true positives and negatives in the green cells and false positives and negatives in the red cells. (b) Performance metrics (sensitivity, specificity and accuracy) of the individual modalities MRI (blue) and EEG (green) as well as of the combined magnetic resonance imaging–electroencephalography markers (yellow)

Table 4 summarizes the results of discriminating the two groups (MMSE ≥ 24 and MMSE < 24) using the same marker subset from MRI and EEG individually, as well as in a modality‐combined manner. Using the latter, individuals were classified with an accuracy of 84.7% (sensitivity 92.1%, specificity 75.0%). Figure 4 visualizes the SVM results in greater detail. The confusion matrix in Figure 4a contains the true positive and negative (green) and false positive and negative (red) elements resulting from the combined MRI–EEG classification, which correctly identified 58 of 63 subjects with MMSE ≥ 24 and 36 of 48 subjects with MMSE < 24. Figure 4b illustrates that the combined MRI–EEG approach yielded better results as compared to the individual modalities. MRI markers separated the groups with an accuracy of only 66.7% (sensitivity 77.8%, specificity 52.1%). EEG markers apparently reflected the cognitive deficits better than MRI, and they separated the groups with an accuracy of 79.3% (sensitivity 87.3%, specificity 68.8%).

**Table 4:** Evaluation of MMSE ≥ 24 and MMSE < 24 classification using magnetic resonance imaging (MRI) markers, electroencephalography (EEG) markers, and markers from both modalities

	Sensitivity (%)	Specificity (%)	PPV (%)	NPV (%)	Accuracy (%)
MRI	77.78	52.08	68.06	64.10	66.67
EEG	87.30	68.75	78.57	80.49	79.28
MRI + EEG	92.06	75.00	82.86	87.80	84.68

Note

NPV: negative predictive value; PPV: positive predictive value.

## 4 DISCUSSION

The first study question sought to determine whether there was a significant relation between the MRI–EEG markers and the severity of cognitive impairment in early AD. Prior studies have reported that individual MRI measures of regional volume and cortical thickness as well as abnormal EEG patterns are related with the cognitive status. Here, only few individual markers were significantly correlated with MMSE scores. However, the combined MRI–EEG markers were well associated with the subjects’ cognitive status. By measuring the left temporal volume, the cortical thickness of frontal, parietal and occipital lobe, and the spectral power in the theta and alpha2 frequency bands, 38.2% of MMSE variation was explained. In line with previous studies, the inclusion of age, sex, and education improved these results (Crum et al., 1993; Kittner et al., 1986). O'Connor et al. (1989) suggested that also the sociocultural background, though difficult to quantify, should be included. Other potentially influential factors might include medication and lifestyle.

On the question of the diagnostic utility of MRI and EEG markers, a joint MRI–EEG approach demonstrated higher diagnostic accuracy (84.7%) as compared to the individual modalities. Our results are in agreement with previous research, showing AD classification accuracies between 74%–100% for temporal lobe atrophy (O'Brien et al., 2004), 76.9%–81.7% for volumetric measures of medial temporal structures (Bottino et al., 2002), and 87%–99% for measures of cortical volume and thickness (Du et al., 2007). However, considering the small samples of these studies, the results need to be interpreted with caution. Similar results have been found in EEG studies, with EEG markers yielding accuracies from 76% to 85% (Jelic & Kowalski, 2009; Triggiani et al., 2016). Thereby, it seems that the MRI and EEG modalities might be complementary due to an incomplete overlap in subjects regarding MRI and EEG abnormalities (Strijers et al., 1997). This view got further support by a recent study showing superior AD‐control classification accuracy (90%) for combined MRI and EEG markers as compared to the individual modalities as well. These data suggest that MRI–EEG markers have potential as accurate cognitive staging tools.

One interesting finding is that four of the six selected markers were measures of cortical lobe atrophy. Especially, atrophy patterns in the left temporal, frontal, and parietal lobe are in accord with previous studies (Du et al., 2007; Hwang et al., 2016). Hartikainen et al. (2012) also found occipito–parietal cortical thinning in AD. Caution is advised; however, for the cortical thickness, measures are highly inter‐correlated and any conclusion on topographic patterns needs further validation. In contrast to earlier findings, the medial temporal lobe structures hippocampus, amygdala, and entorhinal cortex were not included in the final model. A possible explanation might be the strong innervation of these structures and the cerebral cortex and, consequently, that limbic atrophy was implicitly included through the coarser cortical measures. Another reason might be that the included EEG markers reflected the changes in the limbic structures. It also seems possible that these relatively small brain structures were subject to measurement bias in contrast to the greater cortical lobes. The most plausible explanation, however, is that these brain structures have already been affected long before AD was diagnosed and that, during early AD, they do not vary as much.

Among the abnormal EEG patterns, increased theta and decreased alpha rhythms, commonly referred to as EEG slowing, were the most significant markers. These findings are consistent with previous studies and the role of theta and alpha rhythms in cognitive performance: Klimesch (1999) observed that “upper alpha desynchronization correlates with semantic memory performance whereas theta synchronization correlates with working memory or episodic memory performance in particular.” The same author stated: “Because alpha frequency varies to a large extent as a function of age, neurological diseases, memory performance, brain volume, and task demands, the use of fixed frequency bands does not seem justified”. The current approach of recording the EEG during a memory task and computing markers using individualized frequency bands corroborate these ideas. Surprisingly, neither coherence nor cMI – both measures of functional coupling – were selected into the final set of markers. Especially, changes in EEG coherence have been related to AD progression (Jeong, 2004). There are several possible explanations for this result. On the one hand, coherence and cMI are clearly correlated with other EEG markers and by including those parts of the functional coupling information is implicitly included in our model. On the other hand, our approach of global markers might not be well‐suited to capture the topographic characteristic of functional coupling markers. Finally, the changes in functional coupling during early AD might be too subtle to be used as accurate marker of cognitive decline.

A limitation of this study is the sole use of MMSE scores as measure of the global cognitive status. AD typically impairs cognitive complex domains, the sequence and severity of impairment varies from patient to patient, even more so in the earliest disease stages. Another well‐described issue of the MMSE is its susceptibility to demographic factors such as age and education. In the current study, we tried to overcome this limitation by including demographic information as covariates. However, a single MMSE cutoff to distinguish stages of cognitive impairment is thus problematic, and it is important to bear in mind the possible resulting bias. By using more elaborate cognitive tests that are less sensitive to demographic factors, further research should be undertaken to investigate which markers reflect deficits in which cognitive domain.

## 5 CONCLUSION

The purpose of this study was a systematic assessment of the usefulness of different MRI and EEG measures as symptom‐independent markers of the severity of cognitive deficits in early AD. Our study has demonstrated the potential of a combined MRI–EEG approach by separating subjects with MMSE ≥ 24 from those with MMSE < 24 with an accuracy of 84.7%. These results suggest that the current diagnostic workup might benefit from an inclusion of joint MRI–EEG markers. These markers may represent noninvasive and cost‐effective means to gain information on early AD progression that could be essential in timely treatment and facilitate additional clinical research. Further studies in larger populations are warranted to validate the current results. Longitudinal study designs are particularly relevant to confirm the evident potential of these markers and to estimate their true diagnostic accuracy. Finally, cross‐modality validation against functional–metabolic imaging might further increase the understanding of disease progression.

## CONFLICT OF INTEREST

None declared.

## Note

1 National Institute of Neurological and Communicative Disorders and Stroke and Alzheimer's Disease and Related Disorders Association.
