# Supplementary material for: Neuroimaging markers of global cognition in early Alzheimer's disease: A magnetic resonance imaging–electroencephalography study
Source: Brain Behav. 2018 Dec 27;9(1):e01197. doi: 10.1002/brb3.1197 (PMC6346656; doi:10.1002/brb3.1197)
Supplement: Supplementary file 1 [file BRB3-9-e01197-s001.docx]

# Supplementary Material

## Technical description of EEG markers

### A.1. Spectral measures

Here we assume that an EEG segment ***x*** with *T* time points and M channels has an underlying weakly stationary stochastic process and can be represented by real-valued matrix with dimensions *(T x M)*

$$\boldsymbol{x}=\left( \begin{matrix} \boldsymbol{x}_{1}^{'} \\ \vdots\\ \boldsymbol{x}_{T}^{'} \end{matrix} \right)=\text{ }\left( \begin{matrix} x_{11} & \cdots& x_{1m} \\ \vdots& \ddots& \vdots\\ x_{T1} & \cdots& x_{TM} \end{matrix} \right) \in\text{ }R^{\left( TxM \right)}.$$

Then the (auto-/cross-)covariance matrix *γ(s)* is estimated by

$$\gamma\left( s \right)= \left\{ \begin{matrix} \frac{1}{T-s}\sum_{t=0}^{T-s-1} \left( x_{t+s}-\frac{1}{T}\sum_{i=0}^{T-1} x_{i} \right)\left( x_{t}-\frac{1}{T}\sum_{i=0}^{T-1} x_{i} \right)^{'} \\ {\gamma(-s)}^{'}, s<0 \end{matrix} \right., s\geq0$$

and, informally, describes the similarity between EEG sample at different time points as a function of the time lag *s* between them. Based on *γ(s)* the spectral density *f(λ)* at (normalized) frequency *λ* is estimated as

$$f\left( \lambda\right)= \frac{1}{2\pi}\sum_{u} w(u)\gamma(u)e^{-i\lambda u}$$

where *w(u)* is a lag-window that weights time points with respect to their closeness to the current time point. In this work we chose a so-called Parzen window that ensures the positive semi-definiteness of *f(λ)* at all frequencies (Parzen, 1962). The spectral density estimates the EEG as sum of sine- and cosine-oscillations in the range between zero and half of the sampling rate, here 64 Hz. We can represent *f(λ)* as matrix

$$f\left( \lambda\right)=\left( \begin{matrix} f_{11}\left( \lambda\right) & \cdots& f_{1M}\left( \lambda\right) \\ \vdots& \ddots& \vdots\\ f_{M1}\left( \lambda\right) & \cdots& f_{\mathrm{MM}}\left( \lambda\right) \end{matrix} \right)$$

where the diagonal *(i,i)*-elements represent the auto-spectral density of the *i*th EEG channel and the off-diagonal *(i,j)*-elements represent the cross-spectral density between EEG channels *i* and *j*. For more information on spectral estimation the reader is referred to Tukey (1967) and Brillinger (1981).

*Individual alpha frequency (IAF)*: The IAF is usually visible during rest with eyes closed as major spectral peak in the alpha frequency-range. Some subjects however have no unique peak in this frequency range and a simple spectral maximum is in these cases insufficient to identify the IAF. This is why the spectral center of gravity is often used instead. Formally, we define this peak in the ith channel from the auto-spectral density by

$${IAF}_{i}=\frac{\sum_{\lambda=8}^{13} \lambda f_{ii}(\lambda)}{\sum_{\lambda=8}^{13} f_{ii}(\lambda)}$$

where *λ* is the non-normalized frequency in Hertz.

*Spectral power in a frequency band*: The relative spectral power *SP* in a certain frequency band *b* in the *i*th channel is simply defined as

$${SP}_{i}=\frac{\sum_{\lambda\in b} f_{ii}(\lambda)}{\sum_{\lambda\in[0,64]} f_{ii}(\lambda)}$$

where the denominator is a normalization by the spectral power over all frequencies, here from 0 to 64 Hz.

*Coherence*: The (magnitude-squared) coherence *C* at frequency *λ* between channels *i* and *j* is derived from the cross-spectral density by

$C_{ij}(\lambda)=\frac{\left| f_{ij}(\lambda) \right|^{2}}{f_{ii}(\lambda)f_{jj}(\lambda)}$ .

Coherence values are between zero and one with values close to one indicating a strong linear dependence between the *i*th and the *j*th channel (Brillinger, 1981). Due to the symmetry *C_ij_ = C_ji_*, coherence provides no information on the direction of influence.

### A.2. Information theory

*Auto- and (cross-)mutual information (aMI and cMI)*: Shannon and Weaver (1949) introduced the concept of mutual information for measuring the information content that is transmitted between two systems. Here it measures the amount of information that can be obtained about one EEG channel *x* by observing its time-shifted version *y* (aMI) or by observing another channel *y* (cMI). It is defined as

$$MI\left( x,y \right)=\sum_{x,y} p_{xy}\left( x,y \right)\log\frac{p_{xy}(x,y)}{p_{x}(x)p_{y}(y)}$$

where *p_xy_* is the joint probability distribution and *p_x_* and *p_y_* are the marginal probability distributions. These distributions can be estimated from the empirical signal distributions as seen in a histogram. The aMI provides information on nonlinear couplings.

## Basics on the statistical tools

### B.1. Generalized multiple regression

Multiple (linear) regression is a generalization of linear regression by considering more than one regressors (biomarkers) to explain one outcome variable (MMSE). Some of the regressors can be covariates as well. Formally, the model can be defined as

$$Y_{i}=\beta_{0}+\beta_{1}X_{i1}+\beta_{2}X_{i2}+\cdots+\beta_{p}X_{ip}+\varepsilon_{i}$$

where *Y_i_* is the *i*th observation of the outcome, *X_ij_* is the *i*th observation of the *j*th regressor, the *β_i_* are the regression coefficients (slopes) to be estimated and *ε_i_* is the *i*th model error. It is assumed that the *ε_i_* have a uniform and Gaussian distribution and are independent from the regressors. If the normality-assumption is violated a transformation of the outcome might be helpful. Here we use a log-transformation as so-called link-function that changes the above equation to

${\log Y}_{i}=\beta_{0}+\beta_{1}X_{i1}+\beta_{2}X_{i2}+\cdots+\beta_{p}X_{ip}+\varepsilon_{i}$ .

This is a generalized regression model. To make the estimated slopes *β_i_* comparable to each other we standardize *Y_i_* and *X_ij_* to have a mean of zero and a standard deviation of one.

### B.2. Akaike information criterion

The Akaike information criterion (AIC) is a means for selecting the best regression model and is a trade-off between the goodness of fit of the model and the complexity of the model that is mostly defined by the number of variables included. It is defined as

$$AIC=n\log{(\sigma}_{\varepsilon}^{2})+2k$$

where *n* is the number of subjects, *k* is the number of variables in the model and *σ_ε_^2^* is the variance of the model error. This error should be minimized while keeping the model as simple (small *k*) as possible. By minimizing the AIC a model that is a good trade-off can be found. More information on AIC estimation can be found in Akaike (1973).

### B.3. Support vector machine

Support vector machine (SVM) is a non-linear machine learning approach that can separate more complex data structures in case they are not separable in a linear way. The idea of SVM is to identify a hyperplane with maximum distance from the nearest data points from either group. These data points are called support vectors and they define the shape of the hyperplane. The so-called kernel trick allows the algorithm to fit the maximum-margin hyperplane in a higher-dimensional space and thus a non-linear separation in lower dimensions. There are several SVM parameter that require a training set and the application of the resulting model to a test set to evaluate its performance. A formal description of SVM is here out of scope, the interested reader is referred to Cortes and Vapnik (1995).

## Supplementary references

Akaike H (1973): Information theory and an extension of the maximum likelihood principle. 2^nd^ Intl Symp Inf Th: 267-81.

Brillinger DR (1981): Time series: data analysis and theory. Holden-Day, San Francisco.

Cortes C, Vapnik V (1995): Support-vector networks. Machine Learning 20(3): 273-297.

Parzen E (1962): On estimation of a probability density function and mode. Ann Math Stat 33(3): 1065-76.

Shannon CE, Weaver W (1949): The mathematical theory of communication. Champaign, Il: University of Illinois Press.

Tukey JW (1967): An introduction to the calculations of numerical spectrum analysis. Spectral Anal Time Series 25-46.
